# Supplementary material for: Pre-existing polymerase-specific T cells expand in abortive seronegative SARS-CoV-2
Source: Nature. 2021 Nov 10;601(7891):110–7. doi: 10.1038/s41586-021-04186-8 (PMC8732273; doi:10.1038/s41586-021-04186-8)
Supplement: Supplementary file 1 — Reporting Summary [file 41586_2021_4186_MOESM1_ESM.pdf]

nature research

Corresponding author(s): Mala Maini, Leo Swadling

Last updated by author(s): Oct, 1 2021

Reporting Summary

Nature Research wishes to improve the reproducibility of the work that we publish. This form provides structure for consistency and transparency in reporting. For further information on Nature Research policies, see Authors & Referees and the Editorial Policy Checklist.

Statistics

For all statistical analyses, confirm that the following items are present in the figure legend, table legend, main text, or Methods section.

n/a	Confirmed	
☐	☒	The exact sample size (*n*) for each experimental group/condition, given as a discrete number and unit of measurement
☐	☒	A statement on whether measurements were taken from distinct samples or whether the same sample was measured repeatedly
☐	☒	The statistical test(s) used AND whether they are one- or two-sided<br>*Only common tests should be described solely by name; describe more complex techniques in the Methods section.*
☒	☐	A description of all covariates tested
☐	☒	A description of any assumptions or corrections, such as tests of normality and adjustment for multiple comparisons
☐	☒	A full description of the statistical parameters including central tendency (e.g. means) or other basic estimates (e.g. regression coefficient) AND variation (e.g. standard deviation) or associated estimates of uncertainty (e.g. confidence intervals)
☐	☒	For null hypothesis testing, the test statistic (e.g. *F*, *t*, *r*) with confidence intervals, effect sizes, degrees of freedom and *P* value noted<br>*Give P values as exact values whenever suitable.*
☒	☐	For Bayesian analysis, information on the choice of priors and Markov chain Monte Carlo settings
☒	☐	For hierarchical and complex designs, identification of the appropriate level for tests and full reporting of outcomes
☒	☐	Estimates of effect sizes (e.g. Cohen's *d*, Pearson's *r*), indicating how they were calculated

Our web collection on statistics for biologists contains articles on many of the points above.

Software and code

Policy information about availability of computer code

Data collection	A complete masked alignment was downloaded from the GISAID EpiCoV database on 26/7/2021 together with a GISAID Audacity phylogeny comprising 611,893 accessions. The alignment was subsampled to include 800 of each defined NextStrain phylogenetic clade, as provided by GISAID metadata. For clades containing less than 800 accessions all representatives of that clade were included resulting in a comprehensive sampling over the global phylogeny of 13,785 accessions encompassing the genomic diversity of SARS-CoV-2 to date (Supplementary Table 4, Extended Data Fig. 5).
Data analysis	Software used for data/statistical analysis: FlowJo v.10.7.1; FACSDIVA v9.0; Prism 7.0e and 9.0; Excel v.16.16.09; R version 3.5.3 with RStudio Version 1.0.153 for Mac.<br><br>Custom scripts used to perform the homology searches, heatmap visualisation and permutation testing are hosted on GitHub (https://github.com/cednotsed/tcell_cross_reactivity_covid.git). Correlogram was produced using corrplot in R (https://github.com/taiyun/corrplot). Polyfunctionality was visualised using SPICE (version 6.0) and pestle (version 2.0), available at https://niaid.github.io/spice/. MUSCLE algorithm with default parameters and percentage identity was calculated in Geneious Prime 2020.1.2. Alignment figures were made in Snapgene 5.1 (GSL Biotech).

For manuscripts utilizing custom algorithms or software that are central to the research but not yet described in published literature, software must be made available to editors/reviewers. We strongly encourage code deposition in a community repository (e.g. GitHub). See the Nature Research guidelines for submitting code & software for further information.

Data

Policy information about availability of data

All manuscripts must include a data availability statement. This statement should provide the following information, where applicable:

- Accession codes, unique identifiers, or web links for publicly available datasets
- A list of figures that have associated raw data
- A description of any restrictions on data availability

All data analysed during this study are included in this published article (and its supplementary information files). Genomic data analysed was obtained from the publicly available NCBI Virus database and, following registration, from the GISAID EpiCoV repository (full list and metadata available at: 10.6084/m9.figshare.16607423). The datasets generated during and/or analysed during the current study are available from the corresponding author on reasonable request. Correspondence and requests for materials should be addressed to MKM or LS.

Protein sequences for SARS-CoV-2 ORF1ab (accession numbers: QHD43415.1, NP_828849.2, YP_009047202.1, YP_009555238.1, YP_173236.1, YP_003766.2 and NP_073549.1) and for HCoV (accessions listed in Supplementary Table 1, NCBI Virus using 245 the taxid: 1118 together with accompanying metadata) were downloaded from the NCBI database (https://www.ncbi.nlm.nih.gov/).

Field-specific reporting

Please select the one below that is the best fit for your research. If you are not sure, read the appropriate sections before making your selection.

☒ Life sciences ☐ Behavioural & social sciences ☐ Ecological, evolutionary & environmental sciences

For a reference copy of the document with all sections, see nature.com/documents/nr-reporting-summary-flat.pdf

Life sciences study design

All studies must disclose on these points even when the disclosure is negative.

Sample size

Sample sizes are given for each figure throughout the paper when individual dots are not shown. Power calculations were performed prior to week 16 sub-study sampling to determine the sample size needed to test the hypothesis that HCW with pre-existing T cell responses are enriched in exposed uninfected group at a range of incidence of infection, assuming 50% of cohort had pre-existing T cell responses. Sample sizes of 18-64 per group were estimated. An age, sex and ethnicity matched nested substudy was designed within the larger (n=731) parent study and 129 attended for 16 week sampling including high volume PBMC isolation. Sample size can vary across figure panels depending on which stimulations were performed (limited by number of PBMC recovered). Cohort sizes given in Figure 1a.

Data exclusions

Classification of HCW and study participants into cohorts is defined in methods as are any specific exclusions of data points from individual graphs. Two HCW in the seronegative cohort (negative for NP and S1 antibodies wk 0-16) had nAb titres just above the threshold IC50 of 50 were excluded from further analyses (exclusion criteria not pre-established, determined using unexposed pre-pandemic and PCR+ samples). No other HCW or individual samples were excluded after data was generated.

Replication

Replication for each assay are described in the methods. Briefly, per sample unstimulated controls were run in duplicate for ELISpot data with no data excluded due to outliers. Due to limited sample availability ELISpots were only repeated on a small number of pre-pandemic samples. Replicates were successful. CTV proliferation assays were repeated and experimental replicates performed on a subset of individuals successfully. Duplicates were used for S1 ELISAs with no outliers excluded. qPCR was repeated on a subset of individuals successfully. Neutralization assays were preformed over a wide range of dilutions in duplicate.

Randomization

Experiments were performed with protocols optimised to reduce batch variation and to ensure mixing of experimental groups across batches e.g Flow cytometer parameters were consistent between runs (No MFI comparisons were performed, only gating and percentage of parent). Samples from pre-pandemic, seronegative HCW and seropositive HCW were ran in parallel on ELISpot plates.

Laboratory-confirmed infection was determined by weekly nasopharyngeal RNA stabilizing swabs and reverse transcriptase polymerase chain reaction (RT-PCR; Roche cobas SARS-CoV-2 test, Envelope [E] gene) and antibody assay positivity (Spike protein 1 IgG Ab assay, EUROIMMUN) and anti-nucleocapsid total antibody assay (ROCHE). The seronegative health care worker group were matched for demographics and exposure to the laboratory-confirmed infected group and was defined by negativity by these three tests at all 16 time points as well as negative for neutralising antibodies at week 16 and at selected prior time points as indicated. Unexposed pre-pandemic samples were not matched for demographics (Demographics given in Extended Data Table 1). 'Close-contact cohort' self-identified as having had close contact (household contact or alert by NHS test-and-trace app of close contact with a confirmed case) were divided into seropositive or seronegative (determined by S1 ELISA), Extended Data Table 4.

Blinding

IFNg-ELISpot assays were performed on HCW cohorts prior to unblinding of group (laboratory-confirmed-infection or seronegative). Other experiments were not randomized and the investigators were not blinded to allocation during experiments and outcome assessment, however, experimental set-up and controls ensured accurate replication across technical replicates (see above and methods).

Reporting for specific materials, systems and methods

We require information from authors about some types of materials, experimental systems and methods used in many studies. Here, indicate whether each material, system or method listed is relevant to your study. If you are not sure if a list item applies to your research, read the appropriate section before selecting a response.

Materials & experimental systems

n/a	Involved in the study	
☐	☒	Antibodies
☒	☐	Eukaryotic cell lines
☒	☐	Palaeontology
☒	☐	Animals and other organisms
☐	☒	Human research participants
☐	☒	Clinical data

Methods

n/a	Involved in the study	
☒	☐	ChIP-seq
☐	☒	Flow cytometry
☒	☐	MRI-based neuroimaging

Antibodies

Antibodies used

Detailed information regarding all antibodies and other fluorescent agents used in this study are listed in the methods with manufacturer, clone, and dilution used.
ELISpot - human anti-IFNγ Ab (1-D1K, Mabtech; 10 μg/ml), biotinylated IFN-γ detection antibody (7-B6-1, Mabtech; 1μg/ml).

FACS: Memory B cell Panel: CD3 Bv510 (Biolegend, clone OKT3, 1:200), CD11c FITC (BD Biosciences, clone B-ly6, 1:100), CD14 Bv510 (Biolegend, clone M5E2, 1:200), CD19 Bv786 (BD bioscience, clone HIB19, 1:50), CD20 AlexFluor700 (BD biosciences 2H7, 1:100), CD21 Bv711 (BD biosciences, clone B-ly4, 1:100), CD27 BUV395 (BD biosciences, clone L128, 1:100), CD38 Pe-CF594 (BD biosciences, clone HIT2, 1:200), IgD Pe-Cy7 (BD biosciences, clone IA6-2, 1:100).

FACS: CTV assay: IL-2 PerCp-eFluor710 (Invitrogen, clone MQ1-17H12, 1:50), TNFα FITC (BD bioscience, clone MAb11, 1:100), CD8α BV785 (Biolegend, clone RPA-T8, 1:200), IFN? BV605 (BD biosciences, clone B27, 1:100), IFN? APC (Biolegend, clone 4S.B3, 1:50), CD3 BUV805 (BD biosciences, clone UCHT1, 1:200), CD4 BUV395 (BD biosciences, clone SK3, 1:200), CD154 (CD40L) Pe-Cy7 (Biolegend, clone 24-31, 1:50), MIP-1-β PE (BD biosciences, clone D21-1351, 1:100).

FACS:MHC class I pentamer panel: CD3 BUV805 (BD biosciences, clone UCHT1, 1:200), CD4 BUV395 (BD biosciences, clone SK3, 1:200), CD56 Pe-Cy7 (BD biosciences, NCAM16.2, 1:100), CD8α Alexa700 (Biolegend, RPA-78, 1:200), post-expansion CD19 Bv786 (BD biosciences, HIB19, 1:100).

Validation

All antibodies and MHC class I pentamers were purchased from well established manufacturers and were validated by the vendor for species and target. e.g. BD biosciences, Biolegend, and Invitrogen antibodies are tested in Knock-out/knock-in primary model systems to ensure biological accuracy in ISO 9001 certified facilities. Side-by-side lot comparisons are performed. Details of antibody clones have been included for cross-referencing of manufacturing company specification/validation processes. We further validated antibodies by titration to optimal concentrations and by using positive controls where possible (e.g. using populations known to express a certain marker or by polyclonal stimulation). MHC class I pentamers were tested in HLA-mismatched individuals to assess background staining and on T cell clones expanded with cognate peptide.

Fluorescence minus one stains were used to define gates in Flowjo for all FACS assays . Positive (SARS-CoV-2 laboratory-confirmer infected) and negative controls (unexposed pre-pandemic samples) were included in each run for memory B cell staining. Positive control wells were used in CTV stains to ensure accurate staining of cytokines and CTV staining was checked on day 0 before stimulation. Unstimulated control wells treated as peptide wells (e.g. addition of DMSO) were run per biological sample for CTV proliferation assays and ELISpots (in duplicate for ELISpots) and all data is presented as background subtracted as described in the methods.

Human research participants

Policy information about studies involving human research participants

Population characteristics

Age, sex and ethnicity of cohorts are provided in Extended Data Table 1 and 4, and in detail for the COVIDsortium in Augusto et al Wellcome Open Research 2020. Substudy recruitment for all wk16 data presented was performed on a cohort of seronegative HCW matched for age, sex, and ethnicity with a group of laboratory-confirmed infected HCW.

Recruitment

Recruitment is described in details in Augusto et al Wellcome Open Research and in the methods section. Adult (>18 years) hospital HCWs who were fit and well to attend work in any role and across a range of clinical areas, were invited to participate via hospital email, posters, staff meetings, training sessions and participant information leaflets (see https://covid-consortium.com). No other inclusion or exclusion criteria were considered.The "COVID-19 Immune Protection and Pathogenesis in Healthcare Worker Bioresource" (NCT04318314) uses a prospective cohort design (Figure 1). The study consists of questionnaires and biological samples (blood samples, nasal swabs ± saliva) performed at all visits: baseline, weekly follow-ups for 15 weeks, and visits at 6 and 12 months. An age, sex and ethnicity matched nested sub-study was designed within the larger (n=731) parent study and 129 attended for 16-week sampling including high volume PBMC isolation. For the 'close-contact cohort' medical students previously enrolled in a BCG vaccine trial (UCL Ethics Project ID Number: 13545/001) were invited to participate by email and were re-consented.

Ethics oversight

The COVIDsortium bioresource was approved by the ethical committee of UK National Research Ethics Service (20/SC/0149) and registered on ClinicalTrials.gov (NCT04318314). The cohort of medical students and laboratory staff was approved by UCL Ethics (Project ID Number: 13545/001) and pre-pandemic healthy donor samples were collected and cryopreserved before August 2019 under ethics numbers 11/LO/0421. All subjects gave written informed consent and the study conformed to the principles

of the Helsinki Declaration.

Note that full information on the approval of the study protocol must also be provided in the manuscript.

Clinical data

Policy information about clinical studies

All manuscripts should comply with the ICMJE guidelines for publication of clinical research and a completed CONSORT checklist must be included with all submissions.

Clinical trial registration	ClinicalTrials.gov (NCT04318314)
Study protocol	ClinicalTrials.gov (NCT04318314), Augusto et al Wellcome Open Research 2020.
Data collection	Data collection is described in detail in Augusto et al Wellcome Open Research and in the methods section. The "COVID-19 Immune Protection and Pathogenesis in Healthcare Worker Bioresource" (NCT04318314) uses a prospective cohort design. The study consists of questionnaires and biological samples (blood samples, nasal swabs ± saliva) performed at all visits: baseline, weekly follow-ups for 15 weeks, and visits at 6 and 12 months. Recruitment was initially at St Bartholomew's Hospital, London, UK (400 HCWs recruited between 23rd and 31st March 2020, just before the peak of new daily cases in London, which happened on the 2nd April, with 1,022 new cases confirmed). To improve statistical power for downstream analyses, we expanded the target sample size to n=1,000 and extended recruitment on 17th April 2020 to other local sites: Royal Free NHS Hospital Trust (large teaching hospital with specialist expertise in infectious diseases). Baseline: Participants complete a baseline questionnaire including standard variables related to demographics and exposures. These included occupation, household details, smoking status, physical activity, anthropometry, medical history (including vaccination history, current medication and dietary supplements), occupational exposure (including specific clinical areas and access to/use of personal protective equipment [PPE]), travel history, previous COVID-19 symptoms, proven contact with SARS-CoV-2 infected individuals, and any prior testing for SARS-CoV-2 infection. Follow-up: Following recruitment (baseline visit), if fit and well to attend work, participants would undertake in-person weekly questionnaires using research electronic data infrastructure (REDCap v8.5.22)16 to capture occupational metadata, new SARS-CoV-2 exposure, symptoms and test results, and biosample collection.
Outcomes	Prospective HCW study. Not applicable

Flow Cytometry

Plots

Confirm that:

☒ The axis labels state the marker and fluorochrome used (e.g. CD4-FITC).

☒ The axis scales are clearly visible. Include numbers along axes only for bottom left plot of group (a 'group' is an analysis of identical markers).

☒ All plots are contour plots with outliers or pseudocolor plots.

☒ A numerical value for number of cells or percentage (with statistics) is provided.

Methodology

Sample preparation	Detailed sample preparation is given in methods. All FACS was performed on frozen and thawed PBMC isolated by density gradient separation. Peripheral blood mononuclear cells (PBMC) were isolated from heparinized blood samples using Pancoll (Pan Biotech) or Histopaque®-1077 Hybri-MaxTM (Sigma-Aldrich) density gradient centrifugation in SepMate tubes (StemCell) according to the manufacturer's specifications. Isolated PBMCs were cryopreserved in fetal calf serum (FCS) containing 10% DMSO and stored in liquid nitrogen.
Instrument	BD biosciences LSRII and Fortessa-X20 flow cytometers.
Software	FACS DIVA version 9.0 was used on instrument and exporting .fcs files were analysed in FlowJo version 10.7.1 (TreeStar)
Cell population abundance	PBMC were stained and run without sorting or enrichment.
Gating strategy	Example gating strategy for CTV proliferation and mapping FACS experiments is given in Extended Data Figure 3a. Example plots are given in Fig. 2c, Fig 3g, and Extended Data Fig 3d and Extended Data Fig. 6a. Data is reported as a percentage of lymphocytes/singlets/live/CD3+/CD4+ or CD8+ defining antigen specificity by production of IFNg and CTV dilution. For memory B cell stains example plots are given in Extended Data Figure 1b and details of cutoff and assay validation are given in Jeffery-Smith et al BioRxiv 2021. Gating is described in legends: MBC expressed as a percentage of lymphocytes, singlets, Live, CD3-CD14-CD19 +, CD20+, excluding CD38hi, IgD+ and CD21+CD27- fractions. MHC class I Pentamer gating and example plots in Extended Data Figure 6c.

☒ Tick this box to confirm that a figure exemplifying the gating strategy is provided in the Supplementary Information.
